# Exosomal long noncoding RNA CRNDE-h as a novel serum-based biomarker for diagnosis and prognosis of colorectal cancer

**DOI:** 10.18632/oncotarget.13465

**Published:** 2016-11-19

**Authors:** Tong Liu, Xin Zhang, Shanyu Gao, Fangmiao Jing, Yongmei Yang, Lutao Du, Guixi Zheng, Peilong Li, Chen Li, Chuanxin Wang

**Affiliations:** ^1^ Department of Clinical Laboratory, Qilu Hospital, Shandong University, Jinan, People's Republic of China; ^2^ Department of Anorectal Surgery, Shandong Provincial Traditional Chinese Medical Hospital, Jinan, People's Republic of China; ^3^ Oncology Center, Qilu Hospital, Shandong University, Jinan, People's Republic of China

**Keywords:** exosome, long noncoding RNA, CRNDE-h, colorectal cancer, biomarker

## Abstract

Cancer-secreted long non-coding RNAs (lncRNAs) are emerging mediators of cancer-host cross talk. The aim of our study was to illustrate the clinical significance of the lncRNA CRNDE-h in exosomes purified from the serum of patients with colorectal cancer (CRC). The study was divided into four parts: (1) The exosome isolated methods and lncRNA detected methods which accurately and reproducibly measure CRC-related exosomal CRNDE-h in serum were optimized in preliminary pilot stage; (2) The stability of exosomal CRNDE-h was evaluated systematically; (3) The origin of exosomal CRNDE-h was explorated *in vitro* and *in vivo*; (4) The diagnostic and prognostic value of exosomal CRNDE-h for CRC were validated in 468 patients. In pilot study, our results indicated that exosomal CRNDE-h was detectable and stable in serum of CRC patients, and derived from tumor cells. Then, the increased expression of exosomal CRNDE-h was successfully validated in 148 CRC patients when compared with colorectal benign disease patients and healthy donors. Exosomal CRNDE-h level significantly correlated with CRC regional lymph node metastasis (*P* = 0.019) and distant metastasis (*P* = 0.003). Moreover, at the cut-off value of 0.020 exosomal CRNDE-h level of serum, the area under ROC curve distinguishing CRC from colorectal benign disease patients and healthy donors was 0.892, with 70.3% sensitivity and 94.4% specificity, which was superior to carcinoembryogenic antigen. In addition, high exosomal CRNDE-h level has a lower overall survival rates than that for low groups (34.6% vs. 68.2%, *P* < 0.001). In conclusion, detection of lncRNA CRNDE-h in exosome shed a light on utilizing exosomal CRNDE-h as a noninvasive serum-based tumor marker for diagnosis and prognosis of CRC.

## INTRODUCTION

Colorectal cancer (CRC) is one of the most frequent malignant tumors worldwide, with an estimated 1.2 million new cases and 608,700 deaths annually[1]. As we know, early diagnosis and early treatment can significantly elevate the overall survival rate of CRC patients [2]. To date, colonoscopy examination can provide high diagnostic accuracy, but it is inconvenient and invasive and may give rise to additional complications, which limits it to the second-level investigation. Biomarkers in plasma and serum perform an important capacity to diagnose cancers. However, in the currently blood test, the best biomarker carcinoembryonic antigen (CEA) exhibited low sensitivity and specificity, particularly in the early stage of cancer. Thus, novel and reliable CRC-specific biomarkers that can complement and improve the current CRC diagnostic strategies are urgently needed.

Emerging evidences indicate that long noncoding RNAs (lncRNAs) is defined as transcripts with a minimum length of 200 nucleotides in size and limited protein-coding potential, which deregulated expression are implicated in the tumor formation and progression [3–5]. As lncRNAs play pivotal roles in tumor development or suppression, they might be feasible as tumor biomarkers [6, 7]. Colorectal neoplasia differentially expressed - h (CRNDE-h) was originally identified as an lncRNA which transcribed from the long arm of Chromosome 16 (16q12.2) in CRC tissues. LncRNA CRNDE-h, 1,059 nt in length, comprises five core exons and two highly-conserved sequences within intron 4 and intron 1. The level of CRNDE-h not only is tissue-specific, but also presents a temporal pattern: high expressed in the early development of human, while low even none expressed in adult tissues [8]. Moreover, several studies indicate that lncRNA CRNDE-h showed increased expressions in a number of human cancers, including acute myeloid leukemias, prostate cancer, and glioma tumor [9–11]. In our previous study, we have elucidated that high expressed lncRNA CRNDE-h in CRC tissues is significantly associated with adverse clinical characteristics and suggested that lncRNA CRNDE-h may become clinically useful as a biomarker for CRC [12]. But it cannot be used for clinical screening purposes because of difficulty in getting biopsies of tissue from patients suspected to be CRC. Thus, finding a noninvasive, sensitive, and cost-effective nucleic acid tumor marker for CRC diagnosis has become a major challenge.

Recent studies have demonstrated that RNAs, including mRNAs, microRNAs and lncRNAs, are secreted from tumor cancer cells into body fluids such as blood, urine, milk, and saliva via exosomes [13, 14]. Circulating exosomes carry regulatory RNA molecules and play a role in long distance cell-cell communication[15]. Emerging evidences have shown that the expression of specific exosomal lncRNAs are correlated with the clinicopathological characteristics of cancer and thus may function as meaningful biomarkers [16–18]. In light of these observations, we hypothesize that detection of exosomal CRNDE-h might provide a noninvasive avenue for diagnosis and prognosis of CRC.

In this study, we systematically investigated the existence, stability and source of exosomal CRNDE-h in the serum. Then, using a large, independent cohort of patients with a variety of CRC presentations and control volunteers with normal colonoscopy (NC), hyperplastic polyp (HP), inflammatory bowel disease (IBD) and adenoma, we analyzed the utility of exosomal CRNDE-h as an early, easily measured serum-based biomarker for CRC.

## RESULTS

### Characterization of exosome in serum

**Figure 1:**
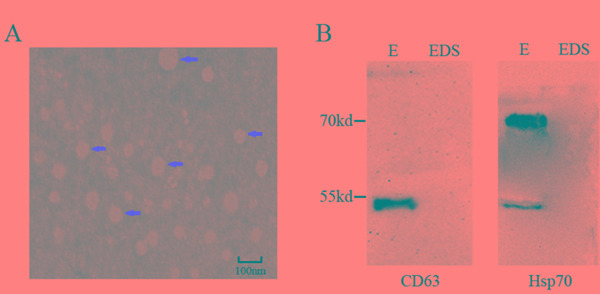
Characterization of serum exosome. **A.** Serum exosomes were analyzed under electron microscopy which displayed the same morphology. Exosomes were highlighted using red arrows. Scale bar = 100nm. **B.** Exosome-enriched protein CD63 and Hsp70 were analyzed by western blotting in exosomal (E) and exosome-depleted supernatant (EDS) samples in serum.

The characteristics of exosomes were determined by transmission electron microscope (TEM) and western blot analysis. Exosome in the serum exhibited double-layer membrane that ranged from 40 nm to100 nm (Figure 1A). In addition, CD63 and Hsp70 which are the protein marker of exosome, were used to confirm the evaluation of exosome by Western blot (Figure 1B).

### Primary data collection

**Figure 2:**
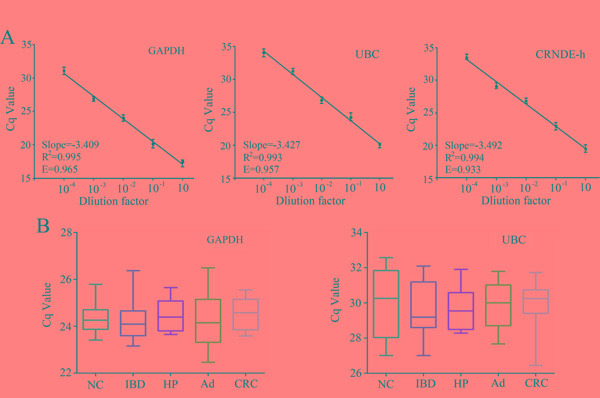
Standard curves and reference gene expression in exosome. **A.** Standard curves for GAPDH, UBC, and CRNDE-h. **B.** Comparison of GAPDH and UBC reference genes expression among NC (n=10), IBD (n=10), HP (n=10), adenoma (Ad; n=10), and CRC (n=10) detected by RT-qPCR. E represents the reaction efficiency.

Melting curve analysis showed that lncRNA CRNDE-h, UBC and GAPDH genes performed a unique peak, respectively. Negative controls showed no detectable Cq value, which verified the lack of any contamination and nonspecific signal. The reaction efficiency of GAPDH, UBC and CRNDE-h calculated from standard curves (Figure 2A) was 0.965, 0.957 and 0.933, which were similar and high, and therefore allowed using the comparative quantification cycle (Cq) method to calculate the relative quantification. Moreover, no significant differences in the expression of exosomal GAPDH and UBC mRNA were found among the five different groups by using RT-qPCR (all at *P* > 0.05) (Figure 2B), indicating that both genes expressed at a constant level in exosomes of serum. Collectively, GAPDH and UBC mRNA could be used as suitable internal controls for normalizing target lncRNA in exosome of serum. Further, we sequenced the amplified product and compared this sequence to a nucleotide database of NCBI using BLAST. Our results indicated that this amplified product was lncRNA CRNDE-h isoform, not others Supplementary Figure S1.

### The general characterization of serum exosomal CRNDE-h

**Figure 3:**
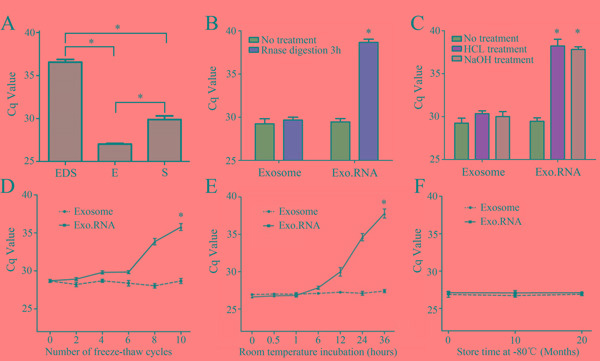
General characterization of the exosomal CRNDE-h. **A.** Exosomal CRNDE-h levels amplified from exosome-depleted supernatant (EDS), serum exosome (E) and whole serum (S). Comparison of the CRNDE-h expression level between exosome group and isolated nucleic acid (Exo.RNA) group when they were subjected to 3 h in RNase A **B.** low (pH = 1), high (pH = 13) pH solutions **C.** multiple freeze-thaws **D.** room temperature incubation **E.** and -80°C **F.** *P<0.01.

Figure 3A showed the average of Cq value for lncRNA CRNDE-h obtained using the same amount of total RNA. RT-qPCR analysis indicated that circulating lncRNA CRNDE-h can be reliably detected in exosomes but hardly detected on the outside circumstance of exosomes. To investigate the potential benefit in using exosomal lncRNA to detect circulating lncRNA CRNDE-h, we directly extracted RNA from serum, skipping the exosome extracted step (whole serum). Data presented in Figure 3A indicated that the Cq value for the exosome samples was lower than the whole serum samples (*P* < 0.01).

To test whether the exosome membrane can protected exosomal CRNDE-h, exosome sample and exosome isolated nucleic acid were left under harsh conditions including incubation of RNase A, acid-base treatment, multiple freeze-thaw cycles, incubation at room temperature, and incubation at -80 °C. Samples were incubated with RNase A, strong acid, and strong base for 3 hours at room temperature, results showed that RNase A (Figure 3B) and acid-base (Figure 3C) treatment had hardly any effect on the level of exosomal CRNDE-h in exosome group, but the isolated nucleic acids group were completely degraded. In freeze-thaw cycles test, our data indicated that the exosomal CRNDE-h showed no significant changes when the exosome group was subjected to ten freeze-thaw cycles, but the isolated nucleic acids group decreased remarkably after ten freeze-thaw cycles (Figure 3D). At room temperature, the expression levels of exosomal CRNDE-h in exosome group was not significantly changed from 0 to 36 hours, but the isolated nucleic acids group markedly decreased after 36 hours treatment (Figure 3E). Moreover, as shown in Figure 3F, exosomal CRNDE-h in both exosome group and isolated nucleic acids group in -80°C treatments remained stable. Collectively, our data indicate that exosomal CRNDE-h are detectable and stable in exosomes, which provides a base factor for CRC diagnosis as the feasible tumor marker.

### Origin of exosomal CRNDE-h in serum

Next, we sought to investigate the hypothesis that serum exosomal CRNDE-h were primarily released or leaked from the tumor cells. Four independent experiments were performed to determine the source of exosomal CRNDE-h in serum.

**Figure 4:**
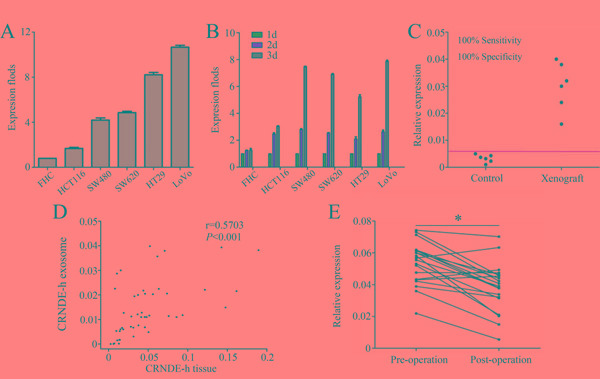
Exosomal CRNDE-h can enter serum at measurable levels Levels of exosomal CRNDE-h expressed in six colorectal cells **A.** and culture medium **B. C.** Levels of exosomal CRNDE-h in xenograft model and controls. **D.** Spearman's correlation analysis between exosomal CRNDE-h levels in tumor tissues and matched serum samples of CRC. **E.** Levels of exosomal CRNDE-h in CRC patients’ serum varied in before surgery (Pre-operation) and 14 days after tumor resection (Post-operation). *P<0.01.

In the first experiment, the expression level of lncRNA CRNDE-h was measured in five CRC cell lines (HCT116, SW620, SW480, HT29, and LoVo), and one normal human intestinal epithelial cell line (FHC) (Figure 4A), and each cell line culture medium which was incubated for 1, 2, and 3 days. Our results demonstrated that exosomal CRNDE-h could enter into the cell culture medium at a detectable level and the expression level steadily elevated over time in five CRC cell lines, but the expression level hardly changed in FHC cell line (Figure 4B).

In the second experiment, xenograft model system was established to investigate whether exosomal CRNDE-h can enter into the circulation at a detectable level. Blood samples were collected after tumors were well established (about 4 weeks after injection). Then we used RT-qPCR methods to assess the exosomal CRNDE-h levels and results illustrated that the presence of tumor can lead to a markedly increase in exosomal CRNDE-h expression in mice serum (Figure 4C).

In the third experiment, lncRNA CRNDE-h expression was measured in serum exosome samples and paired CRC tissues, and then correlation of lncRNA CRNDE-h expression level between the two groups was analyzed. As shown in Figure 4D, a moderate significant correlation was found between lncRNA CRNDE-h amplification in CRC tissue samples and matched serum exosomal CRNDE-h expression (r = 0.570, *P* < 0.001).

The fourth experiment was carried out to compare the exosomal CRNDE-h level in paired pre-operative and post-operative serum samples. Our data showed that the median serum levels of exosomal CRNDE-h were significantly decreased in post-operative samples with 0.040(0.032-0.046) compared with pre-operative samples with 0.056 (0.043-0.062) (*P* = 0.003) (Figure 4E).

### Quantitative analysis of serum exosomal CRNDE-h in the validation phase

**Figure 5:**
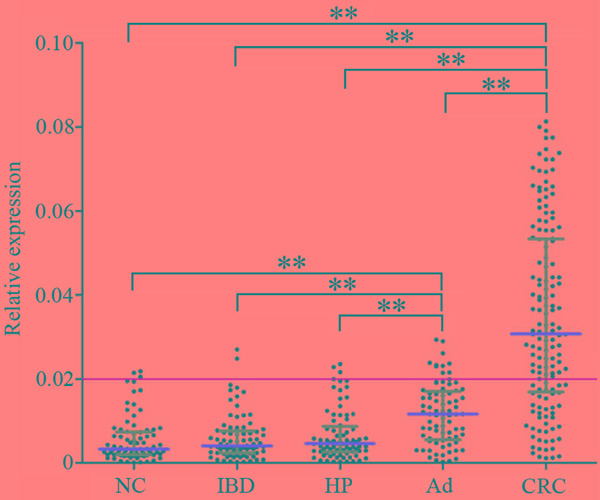
Quantitative analyses of exosomal CRNDE-h in validation phase Relative expression of exosomal CRNDE-h in NC (n=80), HP (n=80), IBD (n=80), adenoma (Ad; n=80), and CRC (n=148). Yellow line represents the optimal cut-off value as 0.020 for discriminating CRC from colorectal benign disease groups and normal colonoscopy. Red line represents the median value and the gray line means the 25% and 75% interquartile range. **P<0.001.

In order to evaluate clinical values of exosomal CRNDE-h in CRC, RT-qPCR method was used to analyze serum levels of exosomal CRNDE-h in an independent large-scale set of samples. Kruskal–Wallis test analysis demonstrated that there was a remarkable difference in exosomal CRNDE-h expression levels among patients with NC, IBD, HP, adenoma, and CRC. The expression levels of exosomal CRNDE-h were significantly elevated in CRC (0.031; 0.017–0.053) compared with NC (0.003; 0.002–0.007), IBD (0.004; 0.002–0.008), HP (0.005; 0.003–0.009), and adenoma (0.012; 0.006–0.017) (all at *P* < 0.001); and its expression levels were also markedly increased in the adenoma compared with NC, IBD, and HP (all at *P* < 0.001) (Figure 5). However, no significant differences were found among NC, IBD, and HP groups (all at *P* > 0.05, respectively).

### Diagnostic performance of exosomal CRNDE-h for CRC

**Figure 6:**
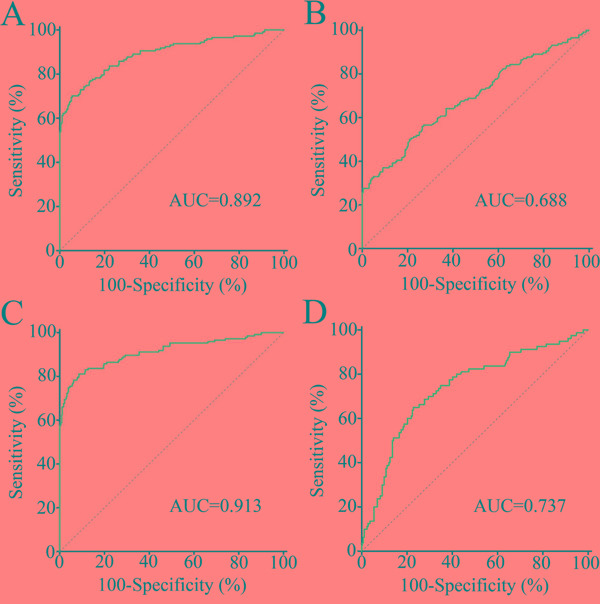
Diagnostic performance of serum exosomal CRNDE-h in CRC ROC curves for detection of CRC using exosomal CRNDE-h **A.**, CEA **B.**, and exosomal CRNDE-h combined with CEA **C.**, as assessed by AUC. **D.** ROC curves for detection of adenoma patients using exosomal CRNDE-h.

Receiver operating characteristic (ROC) curve analyses showed that exosomal CRNDE-h have strong capability for discriminating CRC patients from NC and benign disease group with an area under ROC curve (AUC) value of 0.892 (95% CI: 0.860–0.918, Figure 6A). At an optimal cut-off value of 0.020, the sensitivity and specificity were 70.3% and 94.4%. Conventional tumor marker carcinoembryogenic antigen (CEA) was determined and compared diagnostic power with exosomal CRNDE-h. The AUC for CEA assay was 0.688 (95% CI: 0.644-0.730), and the optimal cut-off value was 5 ng/ml, providing a sensitivity of 37.16% and a specificity of 88.75%, which was markedly lower than that for exosomal CRNDE-h (*P* < 0.001), indicating that exosomal CRNDE-h was superior to CEA in distinguished CRC from colorectal benign disease and NC (Figure 6B). In this study, we further combined CEA with exosomal CRNDE-h, by binary logistic regression, to improve the diagnostic power for CRC. ROC curves analysis illustrated that the AUC for the combination was 0.913 (95% CI: 0.884-0.937, Figure 6C), which was significantly improved compared with exosomal CRNDE-h (*P* = 0.037) or CEA (*P* < 0.001) alone.

Further we used adenoma (n = 80) as the endpoint for detection compared with normal colonoscopy, hyperplastic polyp, and IBD (n = 240), ROC curve analyses indicated that exosomal CRNDE-h yielded an AUC value of 0.737 (95% CI = 0.686–0.785) in identifying patients with adenoma (Figure 6D).

### Correlation of exosomal CRNDE-h with clinicopathological characteristics and survival in patients with CRC

Associations between exosomal CRNDE-h and clinicopathological features of CRC patients were summarized in Table 1. The high expression of exosomal CRNDE-h was significantly correlated with regional lymph nodes metastasis (*P* = 0.019) and distant metastasis (*P* = 0.003). However, no significant correlations were observed between exosomal CRNDE-h over-expression and age, gender, location, size, differentiation, local invasion and CEA level.

**Table 1:** Correlations of clinicopathological parameters and expression level of lncRNA CRNDE-h in patients with CRC (n=148)

Variables	No. of case	CRNDE-h expression Median(interquartile range)	*P* value^a^
Age(years)			0.359
<58	77	0.0282 (0.0180-0.0481)	
≥58	71	0.0330 (0.0158-0.0583)	
Gender			0.141
Male	92	0.0315 (0.0187-0.0560)	
Female	56	0.0282 (0.0118-0.0472)	
Tumor location			0.638
Rectum	97	0.0308 (0.0162-0.0535)	
Colon	51	0.0306 (0.0224-0.0529)	
Tumor differentiation			0.346
Poor	32	0.0428 (0.0169-0.0592)	
Moderate	88	0.0281 (0.0160-0.0475)	
Well	28	0.0311 (0.0177-0.0510)	
Local invasion			0.268
T1-T2	33	0.0249 (0.0097-0.0501)	
T3-T4	115	0.0315 (0.0175-0.0537)	
Regional lymph node metastasis			0.019
Negative	80	0.0257 (0.0165-0.0419)	
Positive	68	0.0397 (0.0185-0.0592)	
Distant metastasis			0.003
No	117	0.0278 (0.0146-0.0472)	
Yes	31	0.0442 (0.0304-0.0608)	
Tumor size			0.059
<5cm	92	0.0277 (0.0142-0.0464)	
≥5cm	56	0.0393 (0.0216-0.0560)	
CEA			0.314
<5ng/ul	93	0.0280 (0.0166-0.0533)	
≥5ng/ul	55	0.0328 (0.0173-0.0554)	

*P*-value was estimated by Mann-Whitney U test.

**Figure 7:**
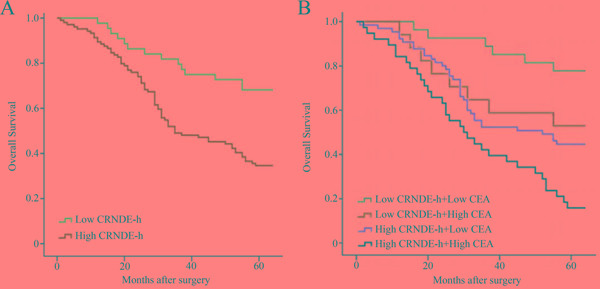
Kaplan–Meier curves for overall survival according to serum exosomal CRNDE-h **A.** and combined exosomal CRNDE-h and CEA **B.** The optimal cut-off value (0.020) of exosomal CRNDE-h was used to categorize the CRC patients into low or high, and low or high CEA based on the cut-off value (5ng/ml).

One-hundred and forty eight patients with CRC were followed up with the mean duration of 44.9 (range 1 – 65) months, and the 5-year overall survival (OS) rate was 45.3%. The CRC patients were divided into two groups (high (n = 104) and low (n = 44)) according to the optimal cut-off value (0.020) of exosomal CRNDE-h level. According to the Kaplan–Meier curve analysis, patients with high serum exosomal CRNDE-h level (34.6%) performed a significantly poorer prognosis than those with low exosomal CRNDE-h level (68.2%) (*P* < 0.001, Figure 7A). To further evaluate the prognostic value of combination of serum exosomal CRNDE-h and CEA, we classified all patients with CRC as low or high serum CEA level based on the standard cut-off value (5ng/ml). Kaplan–Meier survival curve showed patients with both low level of exosomal CRNDE-h and CEA had the highest OS rates, then the decreased survival rates was found in patients with low exosomal CRNDE-h and high CEA level, followed by patients with high exosomal CRNDE-h alone, and the lowest OS rates were observed in patients with both biomarkers in high level (Figure 7B).

We further performed comparisons of the 5-year OS rate with regard to the clinical significance of various prognostic factors, as seen in Table 2. Cox regression univariate analysis revealed a statistically significant correlation between OS and exosomal CRNDE-h over-expression, local invasion, distant metastasis, regional lymph node metastasis, and CEA high-level. More important, according to the univariate analysis data, exosomal CRNDE-h over-expression, local invasion, distant metastasis, regional lymph node metastasis, and CEA high-level were selected and put into the Cox regression multivariate analysis to determine whether exosomal CRNDE-h expression was an independent factor of OS for CRC patients. Our results indicated that only exosomal CRNDE-h over-expression, distant metastasis, regional lymph node metastasis and CEA high-level maintained their significance as independent prognostic factor for OS rates (Table 2).

**Table 2:** Univariate and multivariate cox proportional hazards regression model analysis of overall survival in patients with CRC (n=148)

Variables	Univariate analysis			Multivariate analysis		
	HR	95%CI	*P* value	HR	95%CI	*P* value
Age	1.239	0.804-1.911	0.331		NA	
Gender	1.032	0.661-1.613	0.889		NA	
Tumor location	0.854	0.539-1.354	0.503		NA	
Tumor differentation	0.959	0.670-1.374	0.821		NA	
Local invasion	2.425	1.284-4.581	0.006	1.248	0.635-2.451	0.521
Regional lymph node metastasis	3.701	2.330-5.879	<0.001	3.860	2.360-6.313	<0.001
Distant metastasis	6.209	3.868-9.967	<0.001	5.140	3.097-8.529	<0.001
Tumor size	1.169	1.078-2.584	0.022	1.138	0.725-1.787	0.575
CRNDE-h over-expression	2.724	1.530-4.849	0.001	2.390	1.302-4.386	0.005
CEA high-level	2.054	1.330-3.170	0.001	2.000	1.269-3.154	0.003

**Abbreviations:** HR, hazard ratio; CI, Confidence interval; NA, not considered in the multivariable model.

## DISCUSSION

To the best of our knowledge, this is the first report on investigate the existence, stability and origin of exosomal CRNDE-h in the serum of CRC patients. Then RT-qPCR method was used to test the expression level of exosomal CRNDE-h in patient with a variety of CRC presentations and compared its expression levels to those found in control volunteers with NC, IBD, HP, and adenoma. On the basis of this study, we observed remarkable elevated levels of exosomal CRNDE-h in the serum of CRC, and for the first time demonstrated its potential value in early diagnosis of CRC. Finally, our results indicated that exosomal CRNDE-h might be as a prognostic predictor for CRC.

Diagnostic testing using human blood is considered to be a pleasant, non-invasive and convenient method. Circulating lncRNAs as biomarker for cancer diagnosis have been the subject of extensive research for years, such as POU3F3[19], HOTAIR [20], H19 [21], MALAT-1 [22], and so on. Previously, we have established a novel approach to directly quantify extracellular RNA in serum without RNA extraction [23]. In the current study, we further clarified that the RNA detectable in serum is concentrated in exosomes and purification of exosomes could improves the sensitivity of circulating RNA amplification test. Moreover, we performed a comprehensive study to evaluate the stability of the exosomal CRNDE-h and the results indicate that exosomal CRNDE-h was remarkably stable when treated directly with RNase A digestion and other severe conditions. Thus, we demonstrated that membrane of exosome can protect exosomal CRNDE-h. Furthermore, in this study, we found that exosomal CRNDE-h could enter into the cell culture medium at a measurable level. Subsequently, the xenograft experiment, the correlation of lncRNA CRNDE-h levels between the tumor samples and serum exosome samples matched for the same individuals, and exosomal CRNDE-h assessing between pre-operation and post-operation further indicated that exosomal CRNDE-h was released from tumor cells and can enter into the blood. Thus, the analysis of exosomal lncRNA from blood as a biomarker is more feasible than ever before.

As we know, the development of CRC is considered as a multistep process with the dysregulation of genetics and/or epigenetic resulting in a well-known normal mucosa-adenoma-cancer sequence [24]. Early microarray analysis indicated that knockdown of CRNDE-h with siRNAs resulted in the epigenetic remodeling of chromatin, and particularly in the down-regulation of gene expression via targeted histone methylation or demethylation by PRC2 or CoREST complexes, respectively [25]. Ellis et al. [26] suggested that lncRNA CRNDE-h promotes metabolic changes through insulin/IGF signaling in colorectal cancer cells. Our previous study demonstrated that increased expression of the long non-coding RNA CRNDE-h indicates a poor prognosis in CRC, and is positively correlated with IRX5 mRNA expression [12]. Taken together, these reports suggested that lncRNA CRNDE-h played a pivotal role in CRC formation and development.

Recently, Graham et al [27] detected lncRNA CRNDE-h level in serum of CRC patients using RT-qPCR, and found its level was elevated in serum. The present study purified the exosome and found increased exosomal CRNDE-h in serum of CRC patients when compared to NC, IBD, HP, and adenoma which was consistent with the previous results that found in serum and tissues of CRC [27]. Our study also indicated that exosomal CRNDE-h levels were significantly associated with factors of poor clinical outcome, including positive distant metastasis and positive lymph nodes metastasis. Collectively, these results demonstrated that a higher expression level of exosomal CRNDE-h might be related with an advanced CRC. In addition, the increased expression of exosomal CRNDE-h was also found in the serum of adenoma patients, indicating that increased expression of exosomal CRNDE-h might be an early event in promoting the development of CRC.

We next performed the first investigation of the diagnostic performance of exosomal CRNDE-h for CRC detection. Our results demonstrated that exosomal CRNDE-h could effectively distinguish CRC patients from benign colorectal diseases and NC subjects, with a significantly high AUC value of 0.892, as well as a sensitivity of 70.3% and specificity of 94.4% at optimal cut-off value of 0.020. This data revealed that exosomal CRNDE-h may have superior distinguishing power compared with CEA, a traditional clinical practice circulation tumor marker for CRC. Because there was no significant association between CEA and exosomal CRNDE-h in serum, we further investigated whether detection power could be improved by combination of these serum biomarkers. Intriguingly, when we combined the expressions of the two markers, the AUC was significantly increased, showing a better diagnostic value than that for CEA or exosomal CRNDE-h alone. Therefore, we suggest that detection of combination of exosomal CRNDE-h and CEA might be a feasible complement to current CRC detection strategy. Another interesting finding of our study is that exosomal CRNDE-h showed some diagnosis value for colorectal adenomas. It is important for a CRC early intervention perspective as removal of adenomatous resulted in a reduction of CRC incidence.

In this study, we also estimated the prognostic power of exosomal CRNDE-h through Kaplan-Meier survival analysis. Results indicate that CRC patients with high levels exosomal CRNDE-h showed significantly decreased OS rate than patients with low levels. Taking a step further, univariate and multivariate Cox model analyses showed that exosomal CRNDE-h was an independent prognostic factor as classical prognostic factors like distant metastases, lymph nodes metastasis and CEA. Thus, hypothetically, exosomal CRNDE-h may serve as an accurate biomarker for the prognosis of CRC patients. Moreover, this study demonstrated that the combination of CEA and exosomal CRNDE-h could improve the capability of survival prediction.

To produce reliable data in RT-qPCR assays of target gene, using of appropriate internal reference genes for normalization is a common method [28]. Because there was no information about lncRNAs as references to refer, mRNAs were selected as potential references. In this study, we preliminarily selected two reference genes, GAPDH and UBC, which were stably expressed in serum as described previously [23]. Our results also showed both of them displayed consistent expression in exosome of serum and not influenced by colorectal disease status using RT-qPCR methods. Therefore, GAPDH and UBC were appropriate internal reference for our investigations.

In summary, this is the first study that discovered and proved that exosomal CRNDE-h complies with key characteristics of tumor marker, being non-invasive, performing high sensitivity, specificity, and stableness; moreover, exosomal CRNDE-h is significantly correlated with aggressive tumor behavior and poor prognosis. Therefore, these findings may provide a foundation for development of an early diagnostic and prognostic biomarker for CRC and determination of innovative therapeutic strategies.

## MATERIALS AND METHODS

### Study design, patients, and sample processing

We obtained the written informed consent from each patient and all of the protocols were approved by Ethics Committee of Qilu Hospital, Shandong University. This study was divided into preliminary pilot stage and subsequent validation stage. For pilot study, serum samples were collected from Qilu Hospital of Shandong University to determine the feasibility of the exosomal lncRNA CRNDE-h. In the validation phase, changes of exosomal CRNDE-h in serum were validated in an independent cohort of 468 consecutive hospitalized patients and healthy controls in Qilu Hospital of Shandong University (n=300), and Shandong Provincial Traditional Chinese Medical Hospital (n=168) between July 2007 and February 2010. All individuals were classified into 5 mutually exclusive categories (80 NC, 80 HP, 80 IBD, 80 adenoma, and 148 CRC) based on the histological results of the colonoscopy. Colorectal cancer was staged according to the UICC/AJCC tumor–node–metastasis (TNM) system for CRC. Healthy controls were recruited from people who underwent a routine health checkup and showed no disease. All patients were followed up by clinic visits every 3 months during the first 2 years, every 6 months for 2 years, and every year thereafter until death or March 2015. Follow-up studies included laminography, laboratory tests, and physical examination if necessary. Their medical records, such as sex, age, tumor location, differentiation, tumor size, local invasion, regional lymph nodes metastasis, and distant metastasis were recorded. CRC patients with incomplete medical records, prior chemotherapy or radiation, lost to follow-up, or withdrawal of consent were excluded from this study.

Fifty fresh tumor tissues obtained from CRC patients were macro-dissected within 15 min after resection, confirmed by postoperative pathologic analyses, and then stored at −80°C until RNA extraction.

Blood samples from CRC patients were collected by vena puncture from all subjects before or 14 days after surgery. Cell free serum was isolated using two step centrifugation protocol as we described previously [23], 1600 g for 10 min with another 16,000g for 10 min at 4°C, and then stored at −80°C until exosome extraction. Blood samples with hemolysis were excluded.

### Cell lines and culture

Human CRC cell lines (HCT116, SW480 SW620, HT-29 and LoVo) and human normal intestinal mucosa cell line (FHC) were purchased from the Type Culture Collection of the Chinese Academy of Sciences (Shanghai, China). All cell lines were cultured in RPMI-1640 medium nutrient mixture (Hyclone, Logan, UT) supplemented with 10% fetal bovine serum (Invitrogen, Carlsbad, CA) in a humidified atmosphere containing 95% air and 5% CO2 at 37°C until 90% confluent. Supernatants were collected, centrifuged for 1600 g for 10 min with another 16,000g for 10 min at 4°C, and then stored at −80°C until exosome extraction.

### Isolation of exosome

Serum and medium were filtered through a 0.45-μm pore polyvinylidene fluoride filter (Millipore, Billerica, Mass), subsequently ExoQuick solution (System Biosciences, Mountain View, CA) was added to serum then incubated at room temperature for 0.5 hour, and ExoQuick-TC solution was added to medium then incubated at 4°C for 12 hours, respectively. Exosome was collected by centrifugation at 1500g for 30 minutes. Exosome pellets were resuspended in 25 μl phosphate-buffered saline (PBS).

### Transmission electron microscopy

The sample of exosomes was diluted to 0.5 mg/ml by PBS. Subsequently, the specimen of exosomes was spotted onto a glow-discharged copper grid on the filter paper then was dried for 10 min using the infrared lamp. Finally, exosomes was stained with a drop of 1 % aqueous solution of phosphotungstic acid for 5min then was dried for 20 min using the infrared lamp. Exosomes were examined under transmission electron microscopy (JEM-1-11 microscope, Japan) at 100 keV.

### Western blot analysis

Total protein of exosome was extracted with RIPA buffer (Sigma-Aldrich), and then the protein concentration was measured by BCA assay (Pierce, Rockford, IL). Sodium dodecyl sulfate-polyacrylamide gel electrophoresis and Western blot analyses were performed according to the standard procedures. The membranes and contents were probed using the following antibodies: anti-CD63 antibody (rabbit IgG), and anti-Hsp70 (rabbit IgG) (System Biosciences, Mountain View, CA). Secondary antibodies were goat anti-rabbit HRP secondary antibody (System Biosciences, Mountain View, CA).

### Extraction of total RNA

Total RNA was extracted from tissues/cells using TRIzol reagent (Invitrogen, Carlsbad, CA) according to the manufacturer's instruction and evaluated by NanoDrop spectrophotometer (Thermo Fisher Scientific).

Exosomal RNA was extracted using a miRNeasy Micro Kit (QIAGEN, Valencia, CA, USA). 20 μl of exosome suspension mixed with 700 μl QIAzol lysis buffer, and the mixture was processed according to the manufacturer's standard protocol. The extracted RNA was eluted with 25 μl of RNase-free water. The quantity and quality of the RNA were evaluated by NanoDrop spectrophotometer (Thermo Fisher Scientific).

### Reverse transcriptase quantitative real-time PCR (RT-qPCR)

Extracted RNA (100 ng) was reverse-transcribed to first-strand cDNA using the High Capacity cDNA Reverse Transcription Kit (Takara, Dalian, China). Then, qPCR was performed using Power SYBR Green (TaKaRa, Dalian, China) on a CFX96 Real-Time PCR Detection System (Bio-Rad Laboratories, USA), which was also used for data collection. The thermal cycling program used for quantification was as follows: an initial denaturation step at 95°C for 15 sec, followed by 40 cycles of 95°C for 5 sec and 58°C for 34 sec, with melting curve analysis. Each measurement was performed in triplicate to remove any outliers. Each RT-qPCR run included a calibrator (expression level of CRNDE-h in HT-29 cells) and a negative control lacking cDNA. Relative expression was calculated according to comparative Quantification cycle (Cq) method. The levels of tissue lncRNA CRNDE-h were normalized using GAPDH, as recommended in other studies. And, exosomal CRNDE-h expression was recorded as the ratio against the geometric mean of GAPDH and UBC [29]. Primer sequence was shown in Supplementary Table S1

According to the formula E=10^-1/slope^-1, we used the slope of the standard curve of cDNA to calculate the reaction efficiency (E).

### CEA assay

Serum CEA were measured by chemiluminescent enzyme immune assay with Roche Cobas e601 Analyzer (Roche AG), and the limit of normality were defined as 5 ng/ml according to the manufacturer's instruction.

### CRC xenograft model

5×10^6^ SW480 cells were suspended in a solution of ice-cold 50% basement membrane matrix (BD Matrigel) in PBS. Then injected the cells into six Male BALB/c nude mice (4-week-old) subcutaneously. Six mice were subjected to the mock injections of 200 μl of ice-cold 50% Matrigel in PBS. After 4 weeks, their blood was collected in procoagulant tube by eyeball enucleation then the mice were euthanised. The exosome processing and RNA isolation were the same as those described for human serum.

### Statistical analysis

SPSS 17.0 for Windows (IBM Corporation, Armonk, NY) software programme were used for the statistical analyses. Results were performed as median and interquartile range. The Mann–Whitney U test and Student t-test were applied for comparisons of exosomal CRNDE-h among two groups, and further the difference between more than three groups were performed using the Kruskal–Wallis test. ROC curves and AUC with 95% CI were established to illustrate the diagnostic power of serum biomarkers in CRC using MedCalc 9.3.9.0 (MedCalc, Mariakerke, Belgium). The Youden index (sensitivity+specificity-1) [30] was used to determine the optimal cut-off point of serum exosomal CRNDE-h levels. Logistic regression model was used to combine exosomal CRNDE-h and CEA, and the generated predicted probability value was used for the ROC curve analysis. Survival rate were calculated using the Kaplan–Meier method and comparisons were performed using the Log-rank test. The prognostic value of exosomal CRNDE-h was further verified using the Cox proportional hazards regression model. Two-side *P*<0.05 was considered as statistically significant.

## SUPPLEMENTARY MATERIALS FIGURES AND TABLES


