# Supplementary material for: Exosomal long noncoding RNA CRNDE-h as a novel serum-based biomarker for diagnosis and prognosis of colorectal cancer
Source: Oncotarget. 2016 Nov 19;7(51):85551–63. doi: 10.18632/oncotarget.13465 (PMC5356757; doi:10.18632/oncotarget.13465)
Supplement: Supplementary file 1 [file oncotarget-07-85551-s001.pdf]

Exosomal Long Noncoding RNA CRNDE-h as a novel serum-based biomarker for diagnosis and prognosis of colorectal cancer

SUPPLEMENTARY FIGURE AND TABLE

A

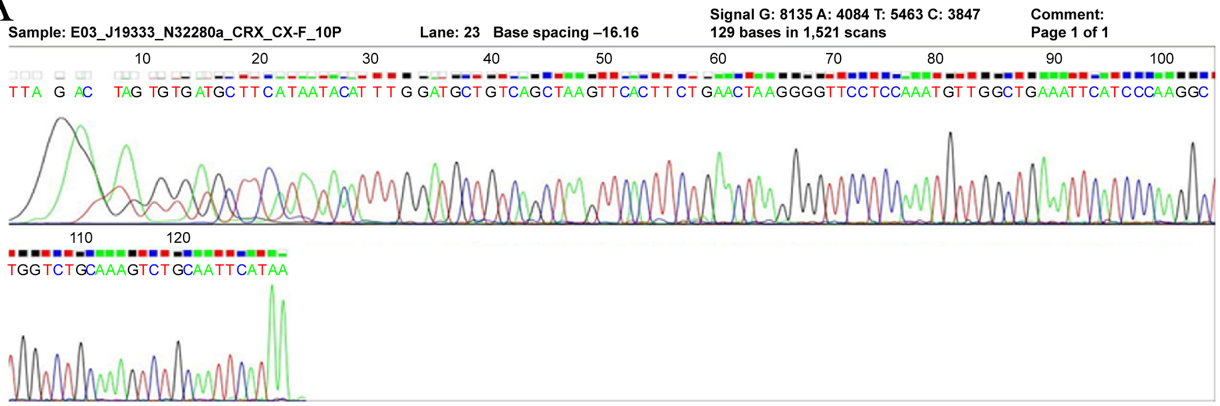

B

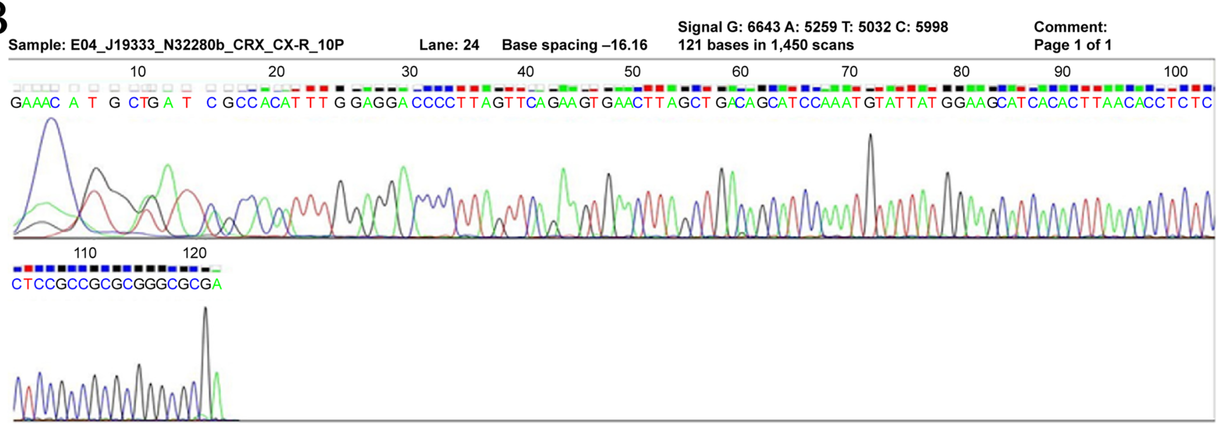

C

```
Query  2    CGCGCCCGCGCGGCGGAGGAGAGGTGTTAAGTGTGATGCTTCCATAATACATTTGGATGC  61
            ||||||||||||||||||||||||||||||||||||||||||||||||||||||||||||
Sbjct  398  CGCGCCCGCGCGGCGGAGGAGAGGTGTTAAGTGTGATGCTTCCATAATACATTTGGATGC  457

Query  62   TGTCAGCTAAGTTCACTTCTGAACTAAGGGGTTCCTCCAAATGTTGGCTGAAATTCATCC  121
            ||||||||||||||||||||||||||||||||||||||||||||||||||||||||||||
Sbjct  458  TGTCAGCTAAGTTCACTTCTGAACTAAGGGGTTCCTCCAAATGTTGGCTGAAATTCATCC  517

Query  122  CAAGGCTGGTCTGCAAAGTCTGCAATTCATAA  153
            ||||||||||||||||||||||||||||||||
Sbjct  518  CAAGGCTGGTCTGCAAAGTCTGCAATTCATAA  549
```

Supplementary Figure S1: Sequencing data of CRNDE-h amplified production. A. The sequencing data of exosomal CRNDE-h amplified production – Forward. **B.** The sequencing data of exosomal CRNDE-h amplified production – Reverse. **C.** Comparison between amplified production sequence and the CRNDE-h sequence in NCBI database.

Supplementary Table S1: Primer sequence

Name	Forword	Reverse
**CRNDE-h**	CGCGCCCGCGCGGCGGAGGA	TATGAATTGCAGACTTTGCAGA
**GAPDH**	GTCAACGGATTTGGTCTGTATT	AGTCTTCTGGGTGGCAGTGAT
**UBC**	ACCCAGCGTACCGGAAGAA	CGGCGTCCACGTCATTTTAT
